# Ampullary Adenocarcinoma with Incidental Pancreatic Neuroendocrine Tumor: Report of an Extremely Rare Case and Review of Literature

**DOI:** 10.7759/cureus.6143

**Published:** 2019-11-13

**Authors:** Matteo Viti, Pietro Maria Lombardi, Mattia Marinelli, Monica Onorati, Corrado D'Urbano

**Affiliations:** 1 General Surgery, G. Salvini Hospital Garbagnate Milanese, Asst Rhodense, Garbagnate Milanese, ITA; 2 Pathology, G. Salvini Hospital Garbagnate Milanese, Asst Rhodense, Garbagnate Milanese, ITA

**Keywords:** ampullary neoplasm, vater’s ampulla adenocarcinoma, pancreaticoduodenectomy, p-nets

## Abstract

Periampullary neoplasms are a heterogeneous group of tumors arising within 2 cm of the ampulla of Vater. Neuroendocrine tumors can originate throughout the entire body, from neuroendocrine cells. These neoplasms exhibit deep differences, according to their origin and biological behavior.

We describe a case of a 79-year-old man who underwent pancreaticoduodenectomy for adenocarcinoma of the ampulla of Vater after proper staging. At gross histology, an incidental pancreatic neuroendocrine tumor was also documented. Despite two synchronous neoplasms, the patient survived 34 months with no evidence of recurrence at follow-up.

The synchronous presence of a second primitive tumor in patients affected by a neuroendocrine tumor is reported in the literature; incidence is variable and the most common site is the gastrointestinal tract. Diagnostic workup for ampullary neoplasms includes abdominal computed tomography (CT) scan, magnetic resonance imaging (MRI) and endoscopic ultrasound (EUS). These investigations infrequently may detect subcentimetric lesions.

We believe this case is currently extremely rare. Preoperative diagnosis of synchronous PanNET would not have changed our approach since surgical therapy represents the gold standard in resectable ampullary neoplasms, and it has a primary role in the prognosis of the present patient.

## Introduction

Periampullary neoplasms are a heterogeneous group of tumors arising within 2 cm of the ampulla of Vater (AoV), including ampullary adenocarcinoma, distal cholangiocarcinoma, duodenal adenocarcinoma, and pancreatic ductal adenocarcinoma [1], having different origins and behaviors. Neuroendocrine neoplasms (NENs) can arise throughout the entire body from neuroendocrine cells; they are often found in the lungs and gastroenteropancreatic (GEP) system, less frequently in the skin, breast, and genitourinary tract [2]. A relationship exists between the presence of GEP-NENs and the simultaneous presence of primitive neoplasms in other body regions [3-4].

To the best of our knowledge, only another one case has been reported in the literature about the synchronous unknown presence of a pancreatic neuroendocrine tumor (PanNET) and AoV adenocarcinoma in the same resected specimen [5]. We herein report one case of a patient affected by these two neoplasms; PanNET diagnosis was performed by gross histology on the resected specimen after duodenopancreatectomy procedure. Moreover, we would like to underline that the detection of PanNET was an occasional finding during the pathological examination of the surgical specimen. This work has been reported in line with the Consensus Surgical Case Report (SCARE) criteria [6].

## Case presentation

A 79-year-old man was admitted to the emergency department of our hospital for abdominal pain and jaundice. He was referred to our attention by the family physician. His medical history consisted of benign prostatic hyperplasia (BPH), mild mitral regurgitation, and chronic obstructive pulmonary disease (COPD), without previous surgical procedures; he had no family history of neoplasms. He was a former smoker. On physical examination, no mass or tenderness was detected. Vital signs were normal. Blood tests showed hyperbilirubinemia (4.5 mg/dl). Abdominal ultrasonography (US) was performed, showing dilatation of both biliary systems.

**Figure 1:**
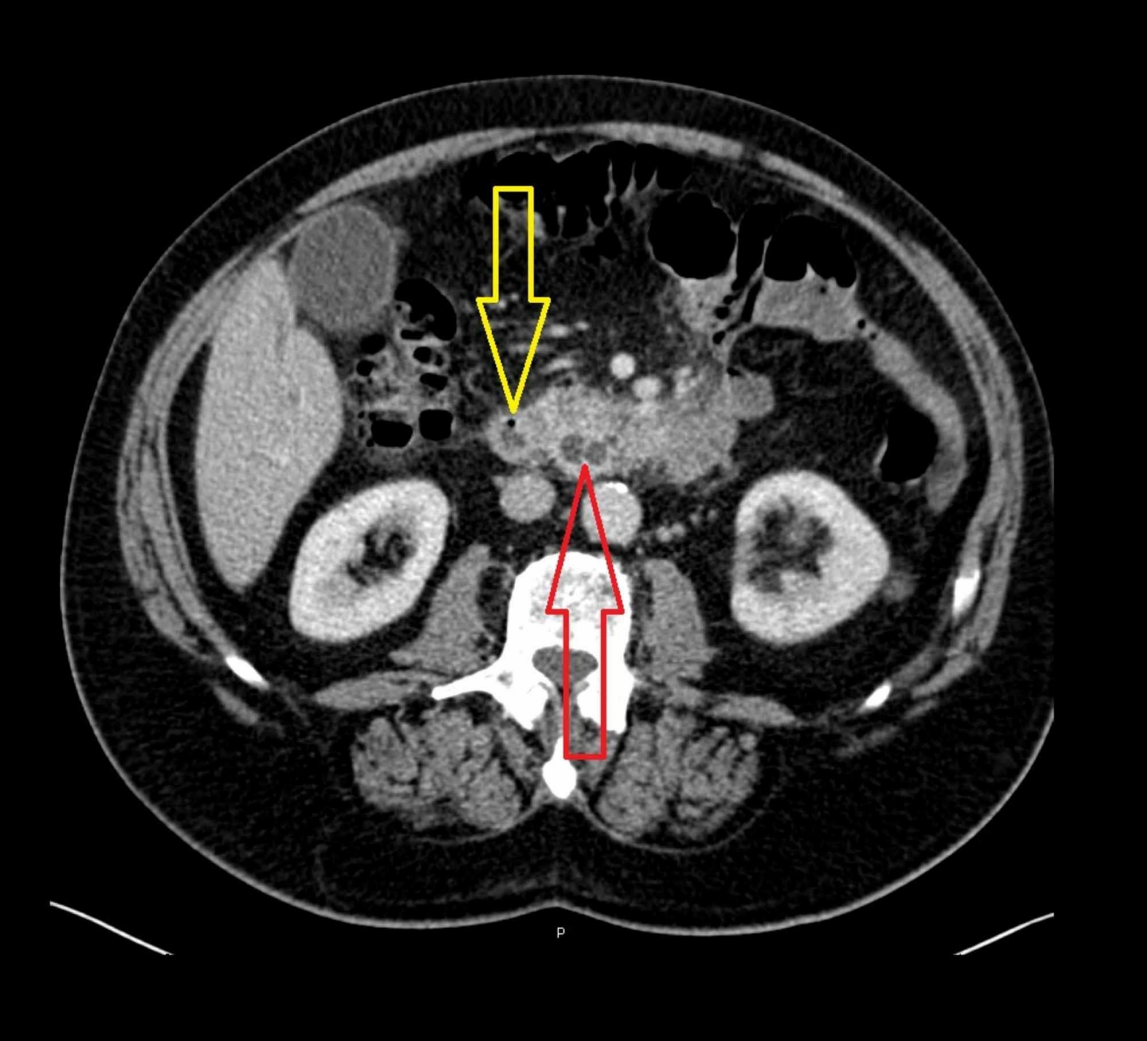
Abdominal CT scan. The red arrow indicates the dilatation of the common bile duct (CBD) and major pancreatic duct (MPD) caused by the endoluminal duodenal mass (yellow arrow). CBD: common bile duct. MPD: major pancreatic duct

The patient was admitted to our department of general surgery in order to perform further diagnostic tests. The abdominal computed tomography (CT) scan (Figure 1) revealed an important dilatation of the biliary system and major pancreatic duct (MPD). The latter and terminal part of the common bile duct (CBD) showed a blunt interruption in the pre-ampullary tract. No other lesions were observed. Tumor markers dosage revealed high levels of CA19.9 (305 U/ml, range 0-33 U/ml).

**Figure 2:**
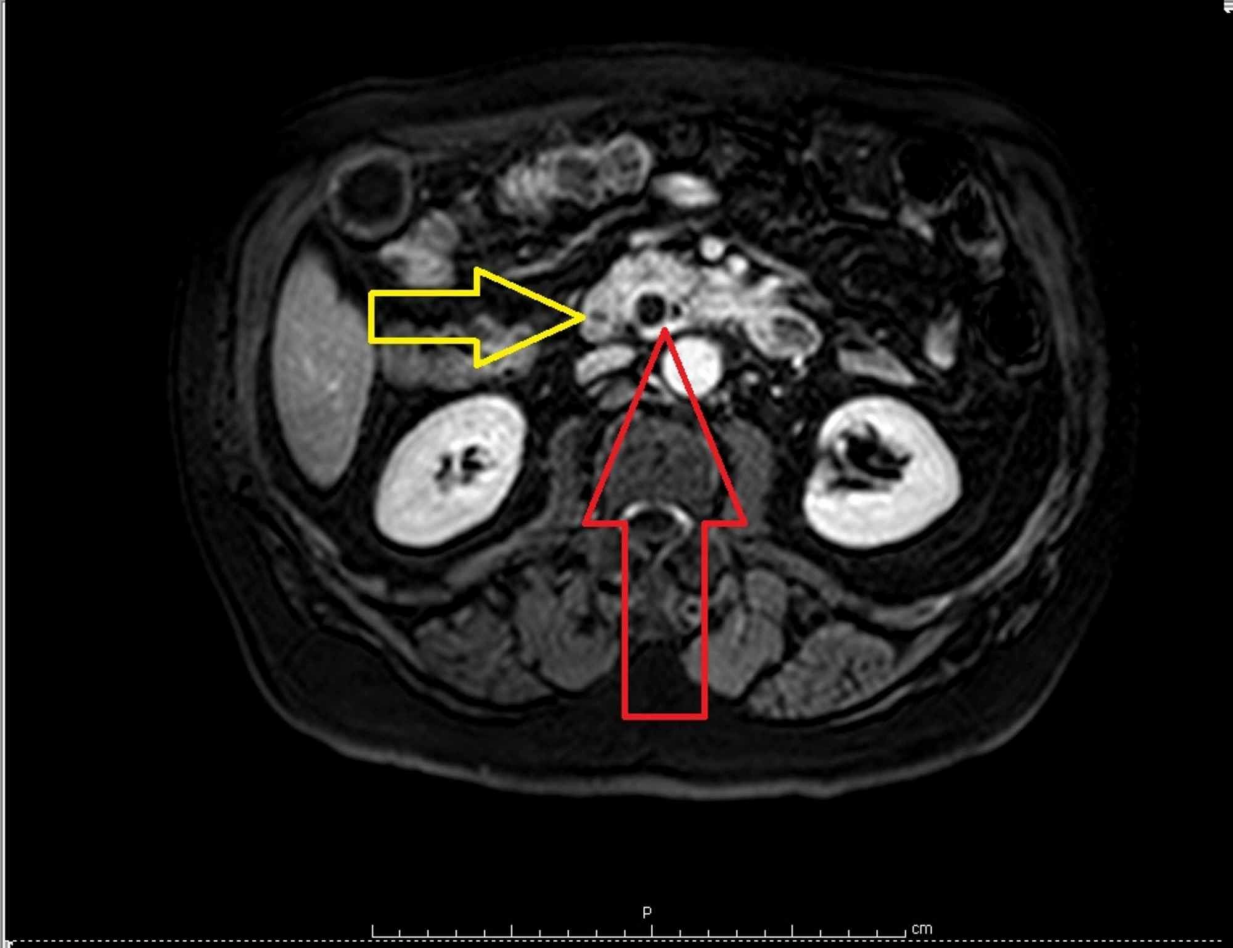
Abdominal magnetic resonance imaging (MRI). The red arrow indicates the dilatation of the common bile duct (CBD) and major pancreatic duct (MPD) caused by the endoluminal duodenal mass (yellow arrow).

**Figure 3:**
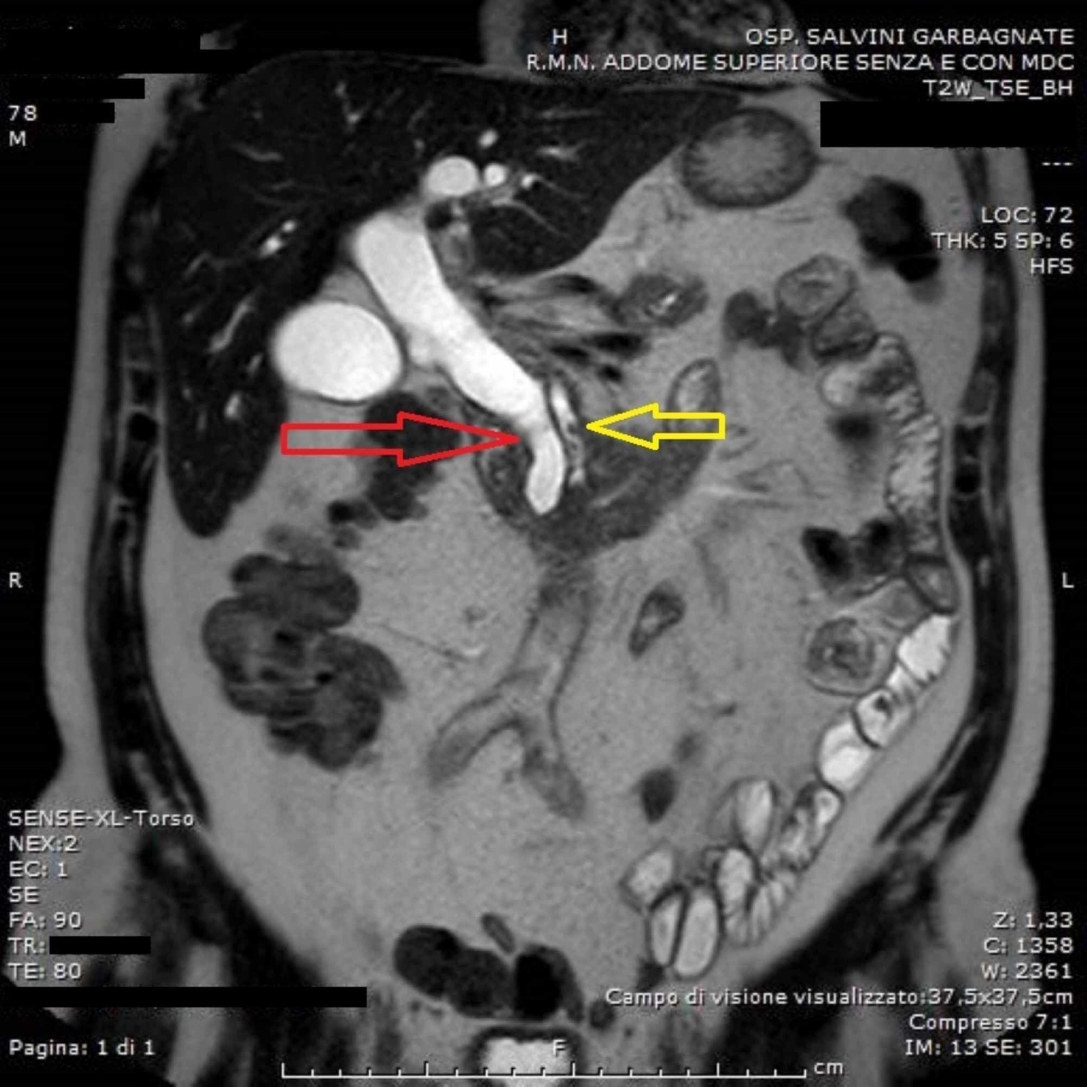
Abdominal magnetic resonance imaging (MRI): dilatation of the common bile duct (red arrow) and the major pancreatic duct (yellow arrow).

Magnetic resonance imaging (MRI) documented a 15-mm low-intensity mass, seemingly protruding in the duodenal lumen with the involvement of the major pancreatic duct (MPD) (Figures 2-3). Due to these findings, a peri-ampullary neoplasm was suspected.

Endoscopic ultrasound (EUS) and endoscopic retrograde cholangiopancreatography (ERCP) confirmed an 18x17 mm ipoechogenic nodular lesion close to AoV, with infiltration of the duodenal muscularis propria and pancreatic parenchyma. An enlarged lymph node was detected at the porta hepatis. During the examination, fine needle aspiration (FNA) of the ampullary lesion was performed, revealing the presence of malignant cells. After a multidisciplinary team meeting, surgery was recommended. A preoperative evaluation was performed, including a chest X-ray, and the patient underwent the Whipple procedure (pancreaticoduodenectomy) with portal and peri-choledocical lymphadenectomy. The postoperative course was uneventful, and the patient was discharged on his eleventh postoperative day in good clinical condition.

**Figure 4:**
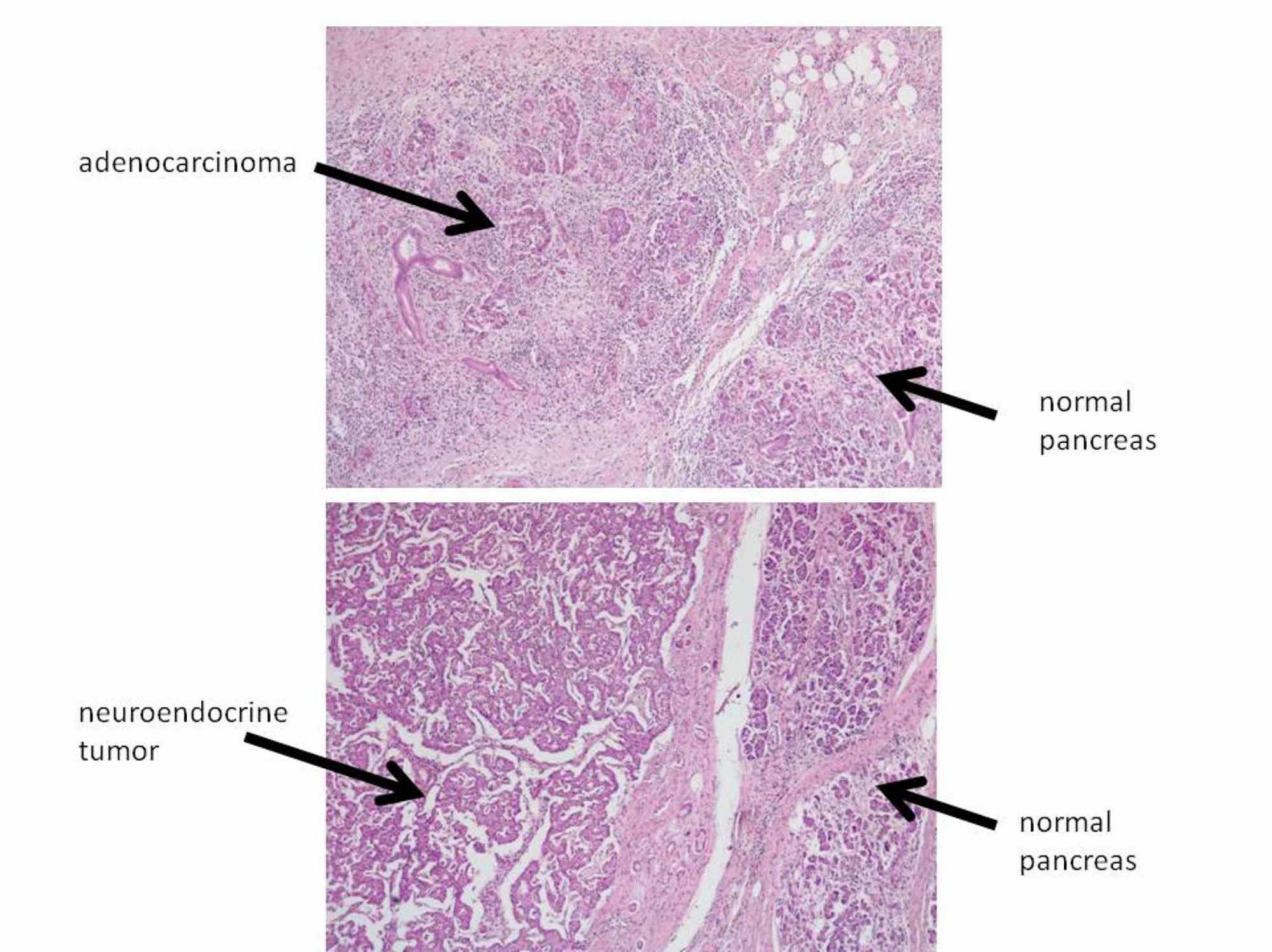
Microscopic examination. In the upper picture, the infiltrating ductal adenocarcinoma shows disordered glandular structures intermingled with mild periglandular fibrosis (H&E; x5). In the lower picture, the neuroendocrine pancreatic tumor shows nests of small polygonal cells, separated by thin fibrous septae and typical salt-and-pepper chromatin in each cell (H&E; x5). H&E: hematoxylin and eosin stain

The macroscopic surgical pathology report described two distinctive neoplasms (Figure 4). The first neoplasm, detected during the radiological examination, was located at the junction of the ampulla and the duodenal mucosa and was described as a 3.5 x 3 cm, grayish, tender, ulcerated mass. The second neoplasm, documented for the first time during the macroscopic examination of the surgical specimen, was described as a 0.7 cm, capsulated lesion, located 1.2 cm away from the ampullary adenocarcinoma, in the lumen of the minor pancreatic duct. Microscopic examination revealed, for the first lesion, an invasive, moderately differentiated, intestinal-type ampullary adenocarcinoma, infiltrating the muscularis propria. The second neoplasm was classified as panNET G2 (Ki67 index: 3% of the neoplastic proliferation) and confirmed by immunohistochemical results (positivity to chromogranin, synaptophysin, and neuron-specific enolase (NSE)). Nine lymph nodes were examined, and all were negative for adenocarcinoma and pNET. The final pathological stage, based on the American Joint Committee on Cancer and the International Union Against Cancer (UICC TNM) staging system (8th edition), was pT2N0Mx G2 for the ampullary adenocarcinoma and pT1N0Mx G2 for the panNET.

The patient survived 34 months after surgical treatment. He died during a hospitalization due to a urinary tract infection. Septic state was the cause of death. No evidence of disease recurrence was found at the last oncological follow-up performed two months prior that hospitalization.

## Discussion

Periampullary neoplasms and neuroendocrine neoplasms (NENs) exhibit significant differences, according to their origin and biological behavior.

The first ones are both benign and malignant lesions that arise at the inlet of the CBD and MPD, in proximity to AoV [2]. AoV adenocarcinoma is a rare lesion, accounting for 0.2% of all malignant neoplasms of the gastrointestinal (GI) tract and almost 6% of periampullary neoplasms [1]. Its incidence is 2.9 new cases per million/year [7]. Diagnosis relies on several clinical, radiological, and endoscopic investigations. Symptoms could include nausea, anorexia, weight loss, and abdominal pain in association with jaundice due to failed bile drainage. An abdominal CT scan allows diagnosis and staging. MRI provides an anatomical representation of the biliary tree and of the main structures of the ampullary region. EUS provides T staging, imaging of surrounding structures, and N staging. ERCP allows sampling and biliary stent placement when appropriate. Surgical resection can be performed with pancreaticoduodenectomy as the standard approach. In high-risk patients, local or endoscopic treatment can be offered [8]. Five-year survival rates are about 10% in pancreatic adenocarcinoma and 40% in AoV adenocarcinoma after adequate resection [7].

NENs develop from neuroendocrine cells, which are located in several organs [2,5]. PanNETs are relatively rare entities, representing almost 1%-2% of whole pancreatic neoplasms [9]. PanNETs can be classified as “functioning” or “non-functioning.” The latter are often asymptomatic and diagnosis is incidental [10] unless mass effect and/or metastatic disease occurs, making surgical cure impossible. The Fifth WHO has classified PanNENs into two groups, well-differentiated (neuroendocrine tumors or NET, G1-2-3) and poorly-differentiated (neuroendocrine carcinoma or NEC, G3) [11].

Preoperative diagnosis can be formulated with high accuracy and sensitivity through EUS, which can detect subcentimetric lesions with mean detection rates of over 90% [12]. Further imaging examinations include an abdominal CT scan, MRI, and somatostatin-analogs-scintigraphy [13]. Surgical resection is mandatory for functioning lesions and for those more of 10 mm because in these two conditions, the risk of lymph node involvement is higher [9,12]. Five-year survival rates are about 30% in non-functioning PanNETs and up to 97% in surgical resectable functioning PanNETs. The synchronous presence of a second primitive tumor in patients affected by GEP-NENs is reported in the literature; incidence is variable from 12% to 46%, and the most common site is the GI tract [3,14-16]. This setting may be explained by “field-effect theory,” whereby a single carcinogenic agent could stimulate the development of both tumors [17-18]. Moreover, NETs produce several neuropeptides/non-neuropeptides comparable to a growth factor and could promote the development of a synchronous primitive tumor within target organs [19]. Therefore, investigating the presence of a synchronous primitive tumor in patients affected by NET is recommended [3].

In the present case, the patient came to our attention with a diagnosis of adenocarcinoma of AoV, and he underwent proper staging by abdominal CT scan, MRI, and EUS. Those investigations did not raise suspicion of any other lesions.

## Conclusions

In conclusion, we believe that the present case is extremely rare since we found only another one case in the literature regarding the synchronous presence of PanNET and AoV adenocarcinoma. Despite proper preoperative workup, PanNET was reported only on histological examination of the surgical specimen. In the present case, according to our experience, a preoperative diagnosis of synchronous PanNET would not have changed our approach. Surgical therapy represented the gold standard treatment and played a primary role in the patient’s prognosis.
